# The negative impact of living environment on intelligence quotient of primary school children in Baghdad City, Iraq: a cross-sectional study

**DOI:** 10.1186/1471-2458-12-562

**Published:** 2012-07-27

**Authors:** Hasanain Faisal Ghazi, Zaleha Md Isa, Syed Aljunid, Shamsul Azhar Shah, Azmi Mohd Tamil, Mohammed A Abdalqader

**Affiliations:** 1 Department of Community Health, Universiti Kebangsaan Malaysia Medical Centre, Kuala Lumpur, Malaysia; 2 United Nations University, International Institute for Global Health, Kuala Lumpur, Malaysia

**Keywords:** Baghdad City, IQ, Living environment, Primary school children

## Abstract

**Background:**

Environmental factors play a very important role in the child development process, especially in a situation like that of Iraq. Thirteen years of economic sanctions followed by the 2003 war and 8 years of unstable security have affected the daily life of Iraqi families and children. The objective of this study was to assess the associations between living environment domains and child intelligence quotient (IQ) score.

**Methods:**

A cross-sectional survey was conducted among 529 children aged 7–8 years from five primary schools in Baghdad during September–October, 2011. The five schools represent people living a range of conditions, and include of both high and low socio-economic groups. Living environment was assessed by 13 questionnaire items, consists of three domains: physical safety , mental stress and public services. While IQ was assessed by Raven Colored progressive matrices.

**Results:**

Among the participants, 22% were of low intelligence versus 77% of high intelligence and 19% lived in a poor environment. There were significant associations between the mental stress and service living environment domains and child IQ (p = 0.009 and p = 0.001, respectively).

**Conclusion:**

In Iraq, child IQ was found to be associated with the mental stress and service domains of the living environment. This study findings will help authorities in their efforts to improve living environment.

## Background

The consequences of war are not limited to impaired physical health. War may also impair the mental health of affected individuals. The negative consequences for mental health can be significant, especially when the victims are children. Living in an unstable environment during childhood has detrimental effects on children, especially on their cognitive development. A few studies have examined the effects of war and terrorism on child cognitive development. Joshi and Donnell [1] concluded that any war or act of terror, as a sudden, unpredictable, and dramatic event, has a tremendous negative impact at various levels, including the community, family, and individual. Children are usually the most affected by these experiences. Delaney-Black [2] examined the relationship between violence exposure, trauma-related distress and standardized test performance among 299 urban first-grade children and their caregivers. Exposure to violence affected the child’s IQ and reading ability. A child experiencing exposure to violence and trauma-related distress at or above the 90th percentile would be expected to have a 7.5 point decrement in IQ and a 9.8 point decrement in reading achievement. Delaney-Black concluded that for young children, exposure to violence and trauma-related distress was associated with substantial decrements in IQ and reading achievement.

During the past three decades, the health of Iraqi people has substantially deteriorated. Since 1980, the country has been caught up in a continuous wave of war and conflict, which has led to a spread of violence and had detrimental effects on people’s living standards. The most recent 2003 war triggered a new wave of disruption in the country [3-5]. The conflict has resulted in an increased incidence of violence and killing, a decline in health services, an unstable electricity supply, a deterioration of drinking water and sewerage systems, a lack of adequate food and major disruption in daily household life [6,7]. It is clear that the recent armed conflict in Iraq has had a profound impact on children’s physical and mental health, but the origins of the problems facing Iraqi children have their roots in the immediate decades prior to 2003. The cumulative effects of this history have left Iraqi children in dire circumstances [8].

This aim of this study was to assess the effect of living in an insecure environment on child intelligence quotients (IQ). We focused on the effects of living in an unstable security situation on the mental health and physical safety of household members and the services provided to the house.

## Methods

A cross-sectional survey was conducted among children aged 7–8 years from five different primary schools in Baghdad City during September–October, 2011. Questionnaires were used to assess the living environment and IQ was assessed using standardized measures. Self-administered questionnaires were distributed to the participating children’s parents.

A list of all schools in the city of Baghdad was obtained from the Ministry of Education. Baghdad is divided into five educational areas, and one school was selected from each of them. Simple random sampling was used to select the five schools. The five educational areas represent people living a range of conditions, and include of both high and low socio-economic groups.

From each selected school, a complete list of student names was obtained. Stratified random sampling according to grade was then used to identify 106 children from each school. To avoid a possible influence of puberty on the measures of interest, children aged 7–8 years were selected as the study population.

G*Power version 3 [9] was used to conduct a power analysis. For 95% power, 0.05 type 1 error (alpha) probability and a correlation coefficient of 0.2 (based on [2]) the minimum sample size required was 266 children.

The Raven’s Colored Progressive Matrices (CPM) test was used to obtain child IQ score, with a maximum total score of 36 points. The test consists of 36 items in three sets of 12: A, AB and B. It is designed for use with young children for anthropological studies and for clinical work. It can be used with people who for any reason cannot speak the English language [10]. Any score higher than the 75^th^ percentile is considered to indicate a high IQ whereas a score below the 75^th^ percentile is considered to indicate a low IQ.

Parents who were illiterate or had primary education (the first stage of compulsory education) were classified as having low education. Parents who had secondary school education (the final stage of compulsory education) or a university degree were classified as having high education. Family monthly income details were obtained from the parents and then categorized into low or high income depending on the median family monthly income published by the Iraqi Central Statistical Bureau [11].

The living environment was defined as the place where the child’s family is living. An insecure living environment was defined as lacking a sense of security or affording no ease or reassurance, wherein the living situation is threatened. The living environment questionnaire consists of three domains: physical safety (hearing any gun shots or explosions, or seeing dead bodies), mental stress (worrying about child safety, child witnessing any type of explosion, thinking of leaving the house and relocating to a different area, thinking the living area is insecure), and public services (water quality, hours of electricity supply).

The questionnaire consists of 13 items, each with three response options: never (2), Occasionally (1) and Frequently (0). The maximum total score was 26. A score of 13 was taken as the cut-point because if the respondents were to answer ‘most of the time’ for all questions, the total score is 13. Any score less than 13 indicated a poor living environment, whereas a score above 13 indicated a good living environment.

A pilot test of the questionnaires was carried out among 31 parents. Back-to-back translation was conducted to validate the questionnaires in Arabic language. Face validity was also tested. The living environment scale had a good internal consistency, with a Cronbach’s alpha coefficient of 0.827.

The study was approved by the Research and Ethics Committee of the Universiti Kebangsaan Malaysia Medical Centre. Also approval from Iraqi ministry of education was obtained to conduct the research inside the schools.

### Statistical analysis

The association between child IQ status and living environment status (good/poor) was examined by a Chi squared test (for categorical variables). A logistic regression analysis was conducted to determine the factors associated with IQ status. Odds ratios were obtained for each potential factor after adjustment for age, sex, school grade, family income and parent education level. The mental stress, physical safety and public services domains were treated as continuous variables. The reference for gender was male, for educational level was high, and for family income was high. All analyses were performed using SPSS version 16.0 [12].

## Results

In total, 529 children aged 7–8 years participated in the study, a response rate of 88.1% (529/600). The participants’ average age was 7.99 years (SD ± 0.55). The IQ scores showed that 77.7% of the participants had a high intelligence level as they scored higher than the 75^th^ percentile. The participants’ median monthly family income was 754,901 Iraqi dinars (approximately 649USD).

Table 1 presents the participants’ socio-demographic characteristics. Slightly more than half (52.2%) were male, and 80.7% had a good living environment.

**Table 1:** Participants’ sociodemographic characteristics by IQ stat

**Variable**	**N**	**low IQ N (%)**	**High IQ N (%)**	**POR(95%CI)**	***P-*** **value**
**Gender**					
Male	276	55 (19.9)	221 (80.1)	0.75 (0.49-1.13)	0.17
Female	253	63 (24.9)	190 (75.1)		
**Mother’s education**					
Low education	92	26 (28.3)	66 (71.7)	1.41 (0.89-2.46)	0.12
High education	433	91 (21.0)	342 (79.0)		
**Father’s education**					
Low education	62	14 (22.6)	48 (77.4)	1.17 (0.61-2.22)	0.62
High education	427	85 (19.9)	342 (80.1)		
**Living environment status**					
Bad	102	52 (51.0)	50 (49.0)	5.66 (3.56-9.08)	<**0.01**^**a**^
Acceptable	427	66 (15.5)	361 (84.5)		
**Family income**					
Low income	267	74 (27.7)	193 (72.3)	1.90 (1.24-2.89)	**0.003**^**a**^
High income	262	44 (16.8)	218 (83.2)		

^**a**^ Pearson chi-squared, significant at p < 0.05.

POR: prevalence odds ratio.

Low education is illiterate or primary school education, high education is secondary or university education.

Table 2 displays the number and proportion of children in each of the two IQ status groups by questionnaire item. Most of the parents (65.2%) reported that the unstable security situation in the last 8 years occasionally affected their family’s daily life, while 22.9% reported that their family’s daily life was frequently affected.

**Table 2:** Associations between living environment variables and child IQ status

**Living environment**	**N**	**Low IQ F (%)**	**High IQ F (%)**	**POR**	***p*** **value**^**a**^
**Mental stress domain**					
**Unstable security situation**					
Frequently	121	29 (24)	92 (76)	1.26	0.33
Occasionally	345	71 (20.6)	274 (79.4)	1.54	
Never	63	18 (28.6)	45 (71.4)		
**Worry about child safety**					
Frequently	171	47 (27.5)	124 (72.5)	0.75	0.11
Occasionally	268	51 (19)	217 (81)	1.21	
Never	90	20 (22.2)	411 (77.8)		
**Child witnessed any type of explosion**					
Frequently	18	9(50.0)	9 (50.0)	0.22	**<0.01**
Occasionally	245	60(24.5)	185 (75.5)	0.69	
Never	266	49(18.4)	217 (81.6)		
**Think of leaving house**					
Frequently	57	15 (26.3)	42 (73.7)	0.68	0.43
Occasionally	258	61 (23.6)	197 (76.4)	0.78	
Never	214	42 (19.6)	172 (80.4)		
**Living area is insecure**					
Frequently	35	14 (40.0)	21 (60.0)	0.36	**0.02**
Occasionally	283	63 (22.3)	220 (77.7)	0.84	
Never	211	41 (19.4)	170 (80.6)		
**Physical safety domain**					
**Hear gun shots**					
Frequently	91	19 (20.9)	72 (79.1)	1.17	0.90
Occasionally	349	78 (22.3)	271 (77.7)	1.07	
Never	89	21 (23.6)	68 (76.4)		
**Hear explosions**					
Frequently	86	16 (18.6)	70 (81.4)	0.98	0.30
Occasionally	345	84 (24.3)	261 (75.7)	0.69	
Never	98	18 (18.4)	80 (81.6)		
**See dead bodies**					
Frequently	6	4 (66.7)	2 (33.3)	0.10	**<0.01**
Occasionally	130	45 (34.6)	85 (65.4)	0.40	
Never	393	69 (17.6)	324 (82.4)		
**Child missed school**					
Frequently	28	16 (57.1)	12 (42.9)	0.20	**<0.01**
Occasionally	321	64 (19.9)	257 (80.1)	1.07	
Never	180	38 (21.1)	142 (78.9)		
**Problem in going to school every day?**					
Frequently	33	21 (63.6)	12 (36.4)	0.11	**<0.01**
Occasionally	253	55 (21.7)	198 (78.3)	0.75	
Never	243	42 (17.3)	201 (82.7)		
**Services domain**					
**Electricity supply**					
< 4 hours per day	110	43 (39.1)	67 (60.9)	0.40	**<0.01**
4 – 8 hours per day	241	38 (15.8)	203 (84.2)	1.40	
> 8 hours per day	178	37 (20.8)	141 (79.2)		
**Water quality**					
Below average	66	22 (33.3)	44 (66.7)	0.40	**<0.01**
Average	242	59 (24.4)	183 (75.6)	0.62	
Good	221	37 (16.7)	184 (83.3)		
**Problems in getting food**					
Frequently	19	9 (47.4)	10 (52.6)	0.24	**<0.01**
Occasionally	213	56 (26.3)	157 (73.7)	0.60	
Never	297	53 (17.8)	244 (82.2)		

^a^ Pearson chi-squared test.

POR: prevalence odds ratio.

There were significant associations between child IQ status and seeing dead bodies in the street, child witnessing any type of explosion, problems attending school every day, child missing school because of an unstable security situation, electricity supply to the house, problems with grocery shopping, water quality and insecure living area.

**Figure 1:**
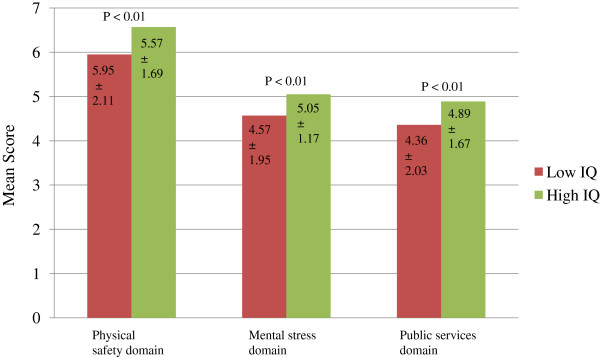
**Association between living environment domain and child IQ status.** Note: Higher scores indicate more favorable conditions. The figure represent the association between environmental domains ( physical safety , mental stress and public services) with child’s IQ level. The three bars represent the domains mean score in low and high IQ level groups. The values inside the bars represent mean ± standard deviation. Independent *t* test was performed to assess the association between the three domains and IQ and it appeared to be significant with p value of < 0.01 respectively. The higher the score of the three domains the better the IQ, in mental stress domain the higher score represent less stress, same things for the other two domains.

Figure 1 displays the mean living environment domain scores (physical safety, mental stress and services provided to the house) by child IQ status. There were significant differences in the mean scores by child IQ status (p < 0.01).

Table 3 presents the results of the multiple logistic regression analysis. After adjusting for gender, parent education level and monthly family income the living environment, in particular, the mental stress and public service domains, still appears to be the most important factor influencing child IQ.

**Table 3:** Summary of multiple logistic regression analysis of factors associated with low IQ status

**Variable**	**B**	**Wald**	***p*** **value**^**a**^	**Adj OR**	**95% CI**
Gender	0.10	0.27	0.59	1.10	0.76-1.60
Family income	0.18	0.72	0.39	1.19	0.79-1.81
Mother education	0.21	0.55	0.45	1.23	0.70-2.18
Father education	−0.17	0.29	0.58	0.83	0.44-1.58
Physical domain	−0.07	1.01	0.314	0.93	0.80-1.07
Mental domain	0.15	5.21	**0.02**	1.17	1.02-1.34
Services domain	0.22	9.98	**<0.01**	1.24	0.95-1.54

^**a**^ Binary logistic regression.

Adj OR: Adjusted odds ratio.

CI: Confidence interval.

## Discussion

The mental stress and public service domains were the two most important living environment conditions affecting child IQ in Baghdad, Iraq. The physical safety domain was not associated with child IQ. Monthly family income was significantly associated with child IQ, which is in agreement with a number of studies that report significant effects of poverty on children’s cognitive and verbal skills [13-15]. Low income results in less food and nutrients for the child, which will in turn affect their development. In this study, parent education level was not associated with child IQ status, although other studies have found a significant relationship between parent education level and child IQ [16]. The majority of the parents in our study area had a high education level, which may explain this discrepancy.

More than two-thirds of the children’s parents were found to live in a good environment according to our cut-off point. The Centre for Strategic and International Studies [17] stated in their 2010 report that “There are a wide range of indicators that show the level of violence in Iraq has dropped sharply since the 2006 year, the average number of security incidents between September and November 2009 was only half that of the same period in 2008.” In addition, the Oxford Research Group Security Report [18] concluded that most of the available evidence supports the view that security within Iraq has substantially improved, but there is still endemic violence, especially in the capital city of Baghdad.

Mental stress domain scores were correlated with child IQ, suggesting that exposure to mental stress is detrimental to child IQ. The two most important components of this domain were child witnessing any type of explosion and insecure family living area in which they perceive as movement restriction and lacking of daily living needs. According other studies [19,20], intensive and continuous exposure to multiple stressful events in conflict and disaster settings is a major risk factor for negative mental health consequences for children and adolescents. Additionally, Tol et al. [21] noted that exposure to violence is a risk factor for adverse child development outcomes in low-income settings, and that childhood mental health problems are difficult to address within the context of ongoing poverty and political instability.

The services domain, which relates to electricity supply to the house, water quality and problems grocery shopping, was also associated with child IQ. It is already established by previous research [22-24] that the quality of the home environment accounts for significant variance in child academic outcomes. In this study, the physical safety domain was not associated with child IQ. A possible reason for this is the improvement in the security situation in Baghdad, because the numbers of dead bodies on the streets, gun shots and explosions have decreased in the last year. According to the Iraqi body count project [25], which recorded Iraqi violence-related deaths during 2003–2011, there was a dramatic decrease of more than 70% in the number of deaths in the 2010–2011 period compared with 2006–2008.

## Conclusions

In Iraq, child IQ was found to be associated with the mental stress and public service domains of the living environment. We hope that our study results will help the authorities and policy makers in Iraq in their efforts to improve the living situation of Iraqis.

## Competing interests

The authors declare that they have no competing interests.

## Authors’ contributions

HFG: Conceived the study idea, analyzed the data and wrote the manuscript. ZMI: contributed to the idea and discussion. SAJ: contributed to discussion. SAS: contributed to analysis and discussion. AMT: contributed to analysis. MAQ: contributed to the methodology. All authors read and approved the final manuscript.

## Pre-publication history

The pre-publication history for this paper can be accessed here:

http://www.biomedcentral.com/1471-2458/12/562/prepub
